# Supplementary material for: Venomix: a simple bioinformatic pipeline for identifying and characterizing toxin gene candidates from transcriptomic data
Source: PeerJ. 2018 Jul 31;6:e5361. doi: 10.7717/peerj.5361 (PMC6074769; doi:10.7717/peerj.5361)
Supplement: Supplemental Information 6 [file peerj-06-5361-s006.gz › FinalOutput_1E-6/Toxin-like_protein_14_1/finaltree.pdf]

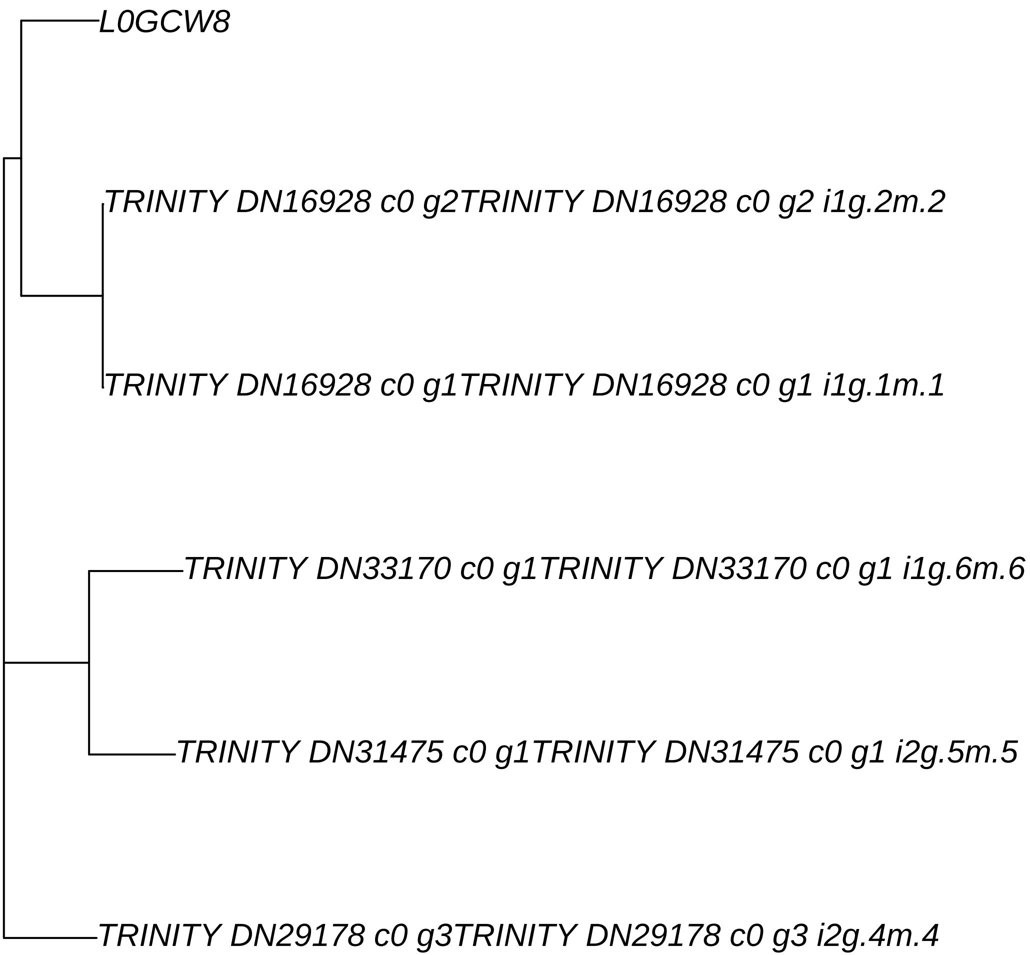
L0GCW8
TRINITY DN16928 c0 g2TRINITY DN16928 c0 g2 i1g.2m.2
TRINITY DN16928 c0 g1TRINITY DN16928 c0 g1 i1g.1m.1
TRINITY DN33170 c0 g1TRINITY DN33170 c0 g1 i1g.6m.6
TRINITY DN31475 c0 g1TRINITY DN31475 c0 g1 i2g.5m.5
TRINITY DN29178 c0 g3TRINITY DN29178 c0 g3 i2g.4m.4
